# Use of plant growth-promoting bacteria to enhance salinity stress in soybean (*Glycine* max L.) plants

**DOI:** 10.1016/j.sjbs.2021.03.053

**Published:** 2021-03-27

**Authors:** Aala A. Abulfaraj, Rewaa S. Jalal

**Affiliations:** a Department of Biological Sciences, Science and Arts College, Rabigh Campus, King Abdulaziz University, Jeddah, Saudi Arabia; b University of Jeddah, College of Science, Department of Biology, Jeddah, Saudi Arabia

**Keywords:** Plant growth promoting rhizobacteria (PGPR), Salt stress, Soybean, Antioxidant enzymes, Protein banding

## Abstract

The effects of three rhizobacterial isolates namely *Pseudomonas fluorescens* (M1), *Pseudomonas putida* (M2) and *Bacillus subtilis* (M3) were examined to enhance growth and chemical components such as chlorophyll and proline of three cultivars of soybean (*Glycine* max L.) under two levels of salinity stress (S1 = 200 mM and S2 = 400 mM of NaCl salt). Several morphological and physiological parameters were investigated. The highest mean values of final germination percent (FGP) were registered in cultivar Crawford (95%) followed by Giza111 cultivar (93%) in the presence of *P*. *fluorescens*, while, FGP of Clark was 85%. Mean germination time was decreased by the application of *P*. *fluorescens* or *P*. *putida* in both salt stressed and unstressed traits. All growth parameters were significantly decreased by salinity treatments, particularly at S2. A significant increase in stem length and shoot fresh weight was recorded in plants treated with *P*. *fluorescens*. This enhancing trend was followed by the application of *P*. *putida* then *B*. *subtilis*. Chlorophyll contents and plant soluble proteins were decreased, while proline content was increased as compared with control treatment. Results showed that the salt tolerant cultivar, Crawford, may have a better tolerance strategy against oxidative damages by increasing antioxidant enzymes activities under high salinity stress. These results suggest that salt induced oxidative stress in soybean is generally counteracted by enzymatic defense systems stimulated under harsh conditions. Our results showed that inoculation with plant growth-promoting rhizobacterial (PGPR) alleviated the harmful effects of salinity stress on soybean cultivars. The diversity in the phylogenetic relationship and in the level of genetic among cultivars was assessed by SDS-PAGE and RAPD markers. Among the polymorphism bands, only few were found to be useful as positive or negative markers associated with salt stress. The maximum number of bands (17) was recorded in Crawford, while the minimum number of bands (11) was recorded in Clark. Therefore, the ISSR can be used to identify alleles associated with the salt stress in soybean germplasm.

## 1 Introduction

Salinity is considered one of the most predominant restraints altering normal growth and metabolism (Kamran et al., 2020). High concentrations of salt in the soil solution and irrigation waters often cause osmotic and ionic stress in plants, resulting in plant toxicity (Hernandez, 2019). It was found that high salinity disturb cellular components such as, cell membranes, proteins, and nucleic acids (Hashem et al., 2019). The cell membrane peroxidation under salinity stress leads to a leakage of vital cellular components that affect plant growth and metabolism (Kamran et al., 2020). The production of toxic radicals (ROS), such as superoxide, peroxides and hydrogen peroxide, causes changes in the normal growth of plants by excess production of peroxyl radicals. Changes in the levels of endogenous phytohormones (such as auxins, gibberellins and cytokinins) under high salt stress were reported to cause toxic effects on plant growth and development (Egamberdieva et al., 2015).

Soybean (*Glycine* max) is an important grain legume crop in many countries. It is considered as a vital source of seed proteins and oil (Hashem et al., 2019). In Saudi Arabia, the mean production of soybean increased substantially from 0 MT in 2005 to 500,000 MT in 2018, and the production of soybean oil reached about 130,000 MT during the same period (United State Dept. of Agriculture, 2019). Salinity stress was found to reduce plant growth, physiological function and yield (Egamberdieva et al., 2015). In soybean there are some chemicals that reflected the genetically controlled resistance features. Molecular and biochemical characters of the tolerance or susceptible cultivars of soybean to salinity stress and the volume of the genetic diversity among cultivars are considering for breeding soybean crop (Omar et al., 2018).

In order to achieve food demands under salinity conditions scientists are involved in finding new approaches for plant stress tolerance. In this regard, Taha et al. (2020) reported that exogenous application of potassium could improve salt tolerance of soybean plants. One friendly solution in this regard is the use of beneficial microorganisms. In this connection, studies based on prediction of plant–microorganism interactions at biochemical, physiological and molecular levels showed that associations with microorganisms largely improved plant responses towards abiotic stress (Lata, 2019). Early studies revealed that some bacteria were found to be associated with some species found in salt affected regions, and these bacteria were known as plant growth promoting bacteria (PGPR) (Sorty et al., 2016). Several studies showed that inoculation with some bacteria can alleviate the harmful effects of salinity stress on the plant (Jochum et al., 2019, Benidire et al., 2020). In an early investigation, Sukweenadhi et al. (2015) found that thale cress (*Arabidopsis thaliana*) inoculated with *Bacillus spp*. and *Micrococcus spp*. were more tolerant than non-inoculated control plants when subjected to different abiotic stresses, mainly salt, drought, and heavy metal stress. To date, a variety of salt-tolerant rhizobacteria including *Rhizobium*, *Pseudomonas* and *Bacillus*, has shown beneficial interactions with many plant species grown in stress conditions (Gopalakrishnan et al., 2015, Jochum et al., 2019). This study hypothesized that increased antioxidant enzymes share in the protection of soybean plants under salt stress condition. Thus, tolerance to salt stress and other stress conditions is vital. This objective can be accomplished through normal conventional breeding with other biotechnology developments to discriminate between various cultivars and choice highly tolerant ones (Saijo and Loo, 2020). The ISSR is one of the most effective molecular tools can be used in this field. It is a quick and easy method for cultivar selection. ISSR markers were used successfully for the assessment of genetic diversity in many crops (Shlvakumar and Subramanya, 2014, Gelotar et al., 2019).

The present study aimed to investigate the effect of some isolates of rhizobacteria, namely *P. fluorescens*, *P*. *putida* and *B. subtilis*, on morphological and physiological characters of three soybean cultivars grown under salinity stress conditions. Molecular markers (ISSR-PCR) connected with salt tolerance in plants were assessed to select salt tolerant cultivars.

## 2 Materials and methods

### 2.1 Plant material

Seeds of three cultivars of soybean (*Glycine* max *L*.) namely Crawford, Giza-111 and Clark were obtained from the Agriculture Extension Dept., Ministry of Environment, Water and Agriculture, the Kingdom of Saudi Arabia and were used in the present study.

### 2.2 Culturing of the microorganisms

The PGPR isolates were supplied by the microbiology Laboratory of the Faculty of Sciences, King Abdulaziz University, Jeddah (KSA). *P. fluorescens* (M1), *P. putida* (M2) and *Bacillus subtilis* (M3) were used in this investigation. The three microorganisms were cultured on broth nutrient culture. A stock solution of each microorganism was made by mixing its culture in a glucose solution (100 mL of 1%) to have a final concentration of 1 × 10^7^ CFU/mL of a specific microorganism.

### 2.3 Salt solution preparation

Solution preparations and water of irrigation required for the present trials was obtained from the Red Sea at the beach of Jeddah town. At this location, salinity level of Red seawater was 36000 ppm (Abdelmongy and El-Moselhy, 2015). Upon diluting seawater, two different salt concentrations [(200 mM (S1) and 400 mM (S2) of NaCl salt] were prepared to be used in the experiments by diluting the sea water using distilled water.

### 2.4 Germination rate (lab. experiments)

Fifty seeds of each soybean cultivar were soaked in 2% sodium hypochlorite for five min to sterilize seeds and then distilled water was used three times to wash the sterilized seeds. The experiment contained 11 treatments for each cultivar and was replicated for 3 times. Treatments were: 1) seeds of each cultivar were immersed in 100 mL of glucose solution (1%) in form of suspension of the microorganism isolates (*P. fluorescens,P. putida*, and *B. subtilis,*) separately and were left at room temperature for 4 h in rate of 1 × 10^7^ CFU/mL. 2) seeds were incubated in 1% glucose solution without microorganisms cells and were sown in sterilized pots filled with wet peat moos (as a control treatment). 3) seeds were then irrigated with the prepared salt solutions (200 or 400 mM) and tap water was used as control, pots were irrigated with 300 mL of salt solution twice a week for each pot. 4). Seeds were inoculated with PGPR in the presence of salt solution. The pots were then positioned in growth chamber at 27 ± 2 °C for one week to estimate the mean germinating rate (MGR) and the final germination percent (FGP) according to the following equations (Moradi et al, 2008):

$$\mathrm{FGP}=\frac{\text{number of germination seeds}}{\text{Total number of seeds}}\times 100$$

Mean germination time (MGT) was calculated according to the following equation (Moradi et al., 2008):

MGT=∑Dn/∑n

where “*n*” is the number of seeds germinated on day “*D*” and “*D*” is the number of days counted from the beginning of germination.

### 2.5 Plant growth (greenhouse experiments)

Soybean seeds were planted in 25 cm diameter. and 30 cm depth plastic pots filled with sand – clay – peat moos (50%: 25%: 25%) in completely randomized design under a greenhouse (Temp. 30 ± 2 °C and 60% RH) for 90 days. In order to ensure proper establishment, the plants were at first irrigated with non-saline water for 21 days. About 2000 mg of NPK (20–20–20) fertilizer (Sangral) was applied/pot fortnightly. As explained above, in the laboratory experiments, greenhouse treatments were replicated 3 times. At harvest time (the end of the experiment), plants were harvested and shoot length and fresh weight were measured.

### 2.6 Biochemical constituents

Chlorophyll pigments (Chl a and Chl b) concentrations were measured spectrophotometerically using 0.1 g of fresh leaves ground in a pre-cold mortar with 6 mL of 80% cold acetone (v/v). The extracted mixture was then filtered and the volume was adjusted to a volume of ten mL with cold acetone solution. The absorbance of the extracts were read at wavelengths of 663 and 646 nm and pigment concentrations were calculated as described by Lichtenthaler (1987).

Soluble protein (mg g^-1^FW) was determined spectrophotometrically as described by Bradford (1976). Briefly, 5 g of fresh leaves were extracted with 5 mL distilled water and 3 mL of Coomassie Brilliant Blue G-250 dye. Sample extract (0.5 mL) was used to measure the absorbance at 595 nm.

As referred by Bates et al. (1973), free proline concentration was determined sing ninhydrin method. Briefly, 5 g of fresh leaf tissues were ground with 3% (w/v) sulfosalicylic acid aqueous solution using a mortar and pestle. The homogenate was filtered through Whatman No.1 filter paper. Then, 2 mL of filtrate was used for the analysis. Two mL of acid ninhydrin and 2 mL of glacial acetic acid were added to the extract. The mixture was then placed in a boiling water bath for one hour, then the reaction was ended by placing the mixture in an ice-filled bath. Four mL of toluene was added to the reaction mixture and the organic phase was extracted by separating funnels. The red color was read spectrophotometrically at 520 nm and toluene was used as blank. The concentration of proline was determined with the aid of calibration curve and was expressed as μg g^-1^FW.

### 2.7 Antioxidant enzymes

One gram of fresh leaf tissues was used to extract the antioxidant enzymes in 5 mL of extraction buffer containing (50 mM K-phosphate buffer, pH 7.6, and 0.1 mM Na_2_-EDTA) with mortar and pestle. The mixture was then centrifuged at 20,000 g at 4 °C for 15 min, and the supernatant fraction was extracted and used to analyze the various enzymes according to Taïbi et al. (2016). Catalase activity (CAT) was determined using reaction buffer mixture containing (25 mM phosphate buffer of pH 7.0, 10 mM H_2_O_2_, and the enzyme extract). The breakdown of H_2_O_2_ was tracked at 240 nm (E = 39.4 mM cm^−1^). The activity of ascorbaic acid peroxidase (APX) was measured spectrophotometrically through measuring the decline in the absorbance of the oxidized ascorbate at wave length 290 nm. One unit of APX was defined as the quantity of the enzyme necessary to consume 1 μmol of ascorbate min^−1^. The activity of glutathione reductase (GR) was assayed via determining the rate of NADPH oxidation caused by the enzyme through following the absorbance of extract at 340 nm. One unit of GR activity was defined as the quantity of enzyme that oxidize 1 μmol of NADPH substrate min^−1^.

### 2.8 Molecular analyses

The extracted proteins were used for the following studies:

a- SDS-PAGE analysis:

Protein profile in leaves of soybean cultivars was assayed by SDS-PAGE Electrophoresis as was defined in earlier study by Laemmli (1970) on a Biorad Protean II system.The technique was carried out as described by Mahgoub et al. (2016). Soybean young leaf tissues (2000 mg) were ground using a pestle and mortar and transferred to Eppendorf tubes (2 mL volume), then 400 µL protein extraction buffer contained (50 mM Tris-HCl of pH 8.0, 0.2% SDS, 500 mM Urea, 1% ß-mercapto-ethanol) was added to the mixture and then was vortexed for 5 min, and the mixture was centrifuged at 4 °C for 10 min at 2400 g to gather the supernatant and remove the remaining residue. The supernatant was collected carefully using a Hamilton syringe. Samples should be cleaned before loading on the gel. In addition, the glass plates were cleaned with 70% ethanol before gel preparation. The gel was placed into an electrophoresis tank and electrode buffer containing (25 mM Tris, 129 mM Glycine, 0.125% SDS) was added. To the mixture, 5 µL protein marker and 10 µL sample were added. The voltage was 180 for 60 min at 100 mA. After electrophoresis gels were picked and located into a tray filled with staining solution composed of (Coomassie Brilliant Blue G-250, methyl alcohol, acetic acid and deionized water) for 30 min then gel was shacked at 40 rpm using electric shaker, and then distained with distaining solution consisted of (Methyl alcohol, acetic acid and deionized water). Gel analysis was performed sing a gel documentation system (Bio-Rad, USA).

b- *ISSR analysis:* DNA of each cultivar was extracted according to Al-Mahgoub et al. (2016). Three primers for ISSR (B04, B06 and B12), synthesized by Bioron Corporation, Germany. were used in this study. PCR was performed in the presence of 50 µg DNA. DNA amplification was done in programmed cycles. An initial denaturation step at 94 °C was made and extension steps were performed at 72 °C for 5 min. PCR amplification products were separated by horizontal gel electrophoreses. DNA marker was applied in the first column of gel. Bands on agarose gel were visualized using UV Transilluminator. Fragments were photographed, using gel documentation equipment, and scored as (+) if present or (-) if absent based on standard marker.

### 2.9 Data analysis

The collected data were statistically analyzed using analysis of variance (ANOVA) as described by Snedecor and Cochran (1980) with the aid of COSTAT computer software. Means were compared by least significant difference (LSD) at 5% levels of probability according to Duncan (1955).

## 3 Results

### 3.1 Germination rate

The effect of salinity and PGPR isolates (M1, M2 and M3) on the FGP and MGT of soybean cultivars were shown in Table 1. The differences in FGP of control treatment between soybean cultivars were insignificant. Both levels of salinity (S1 and S2) resulted in a decrease in FGP of all cultivars. It was clear that S2 treatment was more effective on decreasing FGP than S1 treatment. The lowest value of FGP (60%) was recorded in. Clark cv. under S2 treatment (-31.8), at which Crawford and G-111 showed a decrease in FGP by about 28.6% and 22.2% of control, respectively. In general, salinity treatments caused a significant decrease in FGP values, while the presence of any isolate of the rhizobacteria improved FGP values either in salt treated or untreated plants. In this regard, *B. subtilis* was the best bacterial strain for reducing the deleterious effect of salinity stress on FGP either at S1 or S2. In addition, values of MGT increased with salt treatments, but decreased significantly with rhizobacterial isolates treatments as compared with control. It was clear that Crawford cv. exhibited lower MGT values than other cultivars under both salinity stress and normal condition.

**Table 1:** Effect of two levels of salinity and three isolates of rhizobacteria on final germination percent (FGP) and mean germination time (MGT) of three soybean cultivars.

Soybean cultivars			
Treatments	Crawford	G-111	Clark
Control	91 ± 2.11	90 ± 2.03	88 ± 2.11
S1	73 ± 1.88	75 ± 2.11	68 ± 1.56
S2	65 ± 1.76	70 ± 1.85	60 ± 1.09
M1	94 ± 2.86	91 ± 1.88	88 ± 2.21
M2	94 ± 2.85	93 ± 2.10	86 ± 2.16
M3	96 ± 2.74	95 ± 2.65	90 ± 2.77
S1 + M1	82 ± 2.32	83 ± 2.14	75 ± 2.05
S1 + M2	84 ± 2.08	84 ± 1.89	76 ± 1.68
S1 + M3	87 ± 1.81	85 ± 2.05	79 ± 1.98
S2 + M1	78 ± 2.11	76 ± 2.33	68 ± 1.42
S2 + M2	81 ± 2.45	80 ± 1.87	66 ± 1.82
S2 + M3	87 ± 2.52	81 ± 2.04	70 ± 2.03
LSD at 0.05	0.97	0.75	0.99
	MGT		
Control	2.8 ± 0.11	3.0 ± 0.23	3.1 ± 0.83
S1	3.0 ± 0.68	3.6 ± 0.11	3.7 ± 0.57
S2	2.9 ± 0.76	3.8 ± 0.85	3.9 ± 0.39
M1	2.4 ± 0.46	2.8 ± 0.88	2.7 ± 0.28
M2	2.5 ± 0.55	2.7 ± 0.34	2.8 ± 0.66
M3	2.5 ± 0.74	2.8 ± 0.65	2.6 ± 0.76
S1 + M1	2.5 ± 0.32	3.2 ± 0.44	3.0 ± 0.25
S1 + M2	2.2 ± 0.18	3.4 ± 0.89	3.2 ± 0.61
S1 + M3	3.2 ± 0.55	3.2 ± 0.75	3.2 ± 0.98
S2 + M1	2.9 ± 0.11	3.5 ± 0.73	3.7 ± 0.42
S2 + M2	3.1 ± 0.25	3.7 ± 0.87	3.6 ± 0.82
S2 + M3	3.2 ± 0.53	3.8 ± 0.64	3.1 ± 0.43
LSD at 0.05	0.55	0.32	0.34

Mean of three replicates and ± standard error (*n* = 3), *Pseudomonas fluorescens* (M1)*, Pseudomonas putida* (M2) and *Bacillus subtilis* (M3).

### 3.2 Growth parameters

**Fig. 1:**
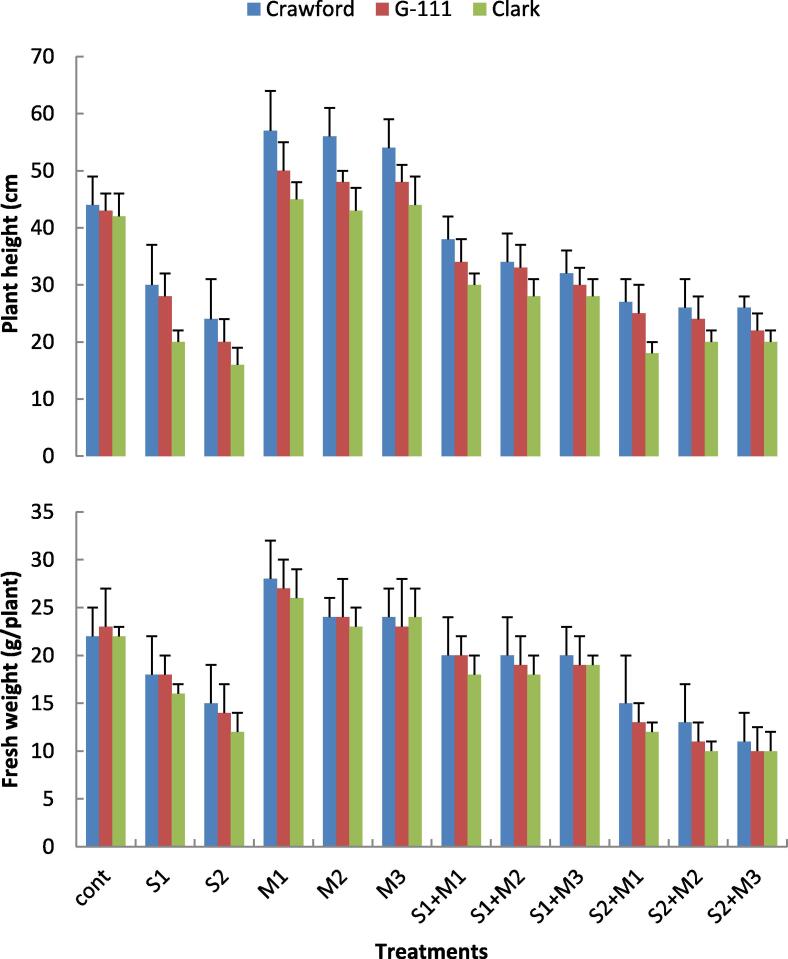
Effects of two salinity levels (S1 and S2) and three rhizobacterial isolates (M1, M2 and M3) on shoot length and shoot fresh weights of three cultivars of soybean.

The effect of salinity stress and PGPR isolates on shoot length and shoot fresh weight were illustrated in Fig. 1. It was clear that the high level of salt stress (S2) was more effective in reducing the values of growth parameters than S1. Data showed that. Clark cv. was the most affected cultivar by salinity treatments. At S2, shoot length decreased by about 45.5, 53.5 and 61.9% of control in Crawford, G-111 and Clark, respectively. Shoot length of salt stressed and unstressed plants increased significantly by rhizobacterial isolates, particularly with *P. fluorescens*. The reduction in shoot fresh weight ranged between 27.4% at S1 and 45.5% at S2. In general data showed that, Crawford cv. seemed to be more tolerant to salinity treatments than the other cultivars. Similar trend was recorded for shoot height. On the other side, all growth parameters were improved in plants treated with any of the bacterial strains, either in the presence or the absence of salt treatments. The maximum values in all the growth parameters were recorded for Crawford cv. inoculated with *P. fluorescens*.

### 3.3 Biochemical constituents

**Fig. 2:**
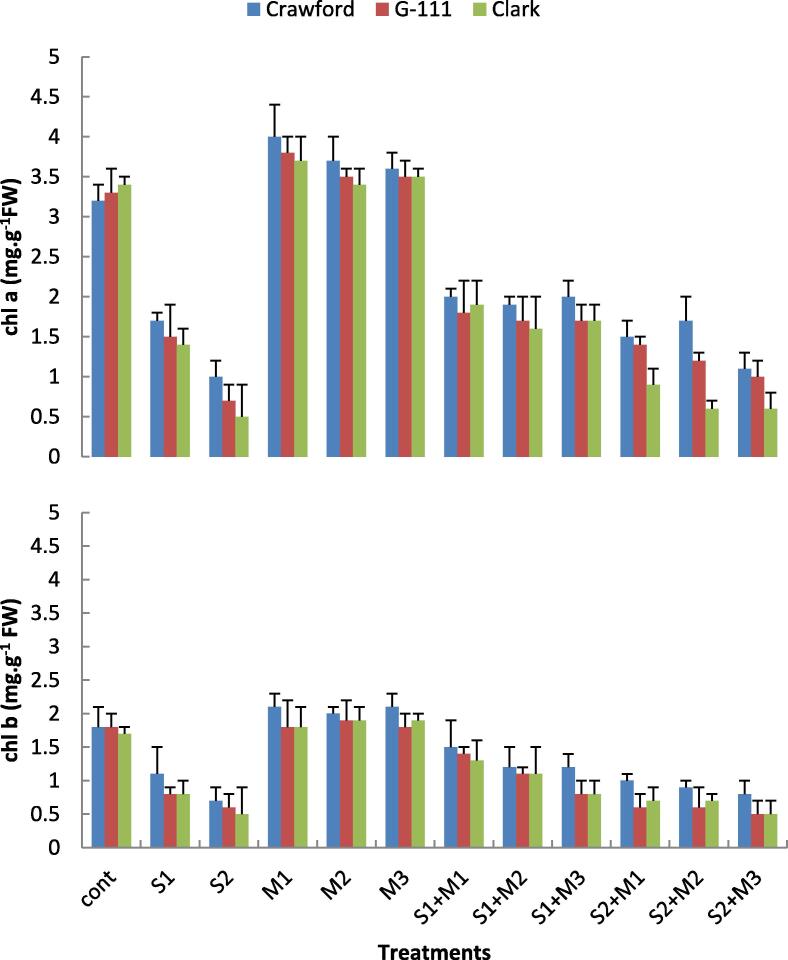
Effects of two salinity levels (S1 and S2) and three rhizobacterial isolates (M1, M2 and M3) on chlorophyll *a* (Chl a) and chlorophyll *b* (Chl b) of three cultivars of soybean leaves.

All salinity concentrations (S1 and S2) caused negative effects on plant chlorophyll content as compared with control treatment. Results showed that an inverse relationship between salinity stress and chlorophyll content, thus S2 was more deleterious on Chl content than S1. At S2 level Chl a contents decreased by about 68.7% in Crawford cv., 78.8% in G-111 cv. and 85.3% in Clark cv.as compared with control. While Chl b content decreased by about 61.1% and 70.6% in Crawford and Clark, respectively, as compared with control. (Fig. 2). All the bacterial isolates improved the chlorophyll content in salt treated or untreated plants as compared with control. In this concern, Chl a in S2-treated Crawford, G-111 and Clark increased significantly as treated with any of the rhizobacterial strains comparing with S2- treated cultivars without rhizobacterial treatments. Chl b followed similar pattern as that of Chl a.

**Fig. 3:**
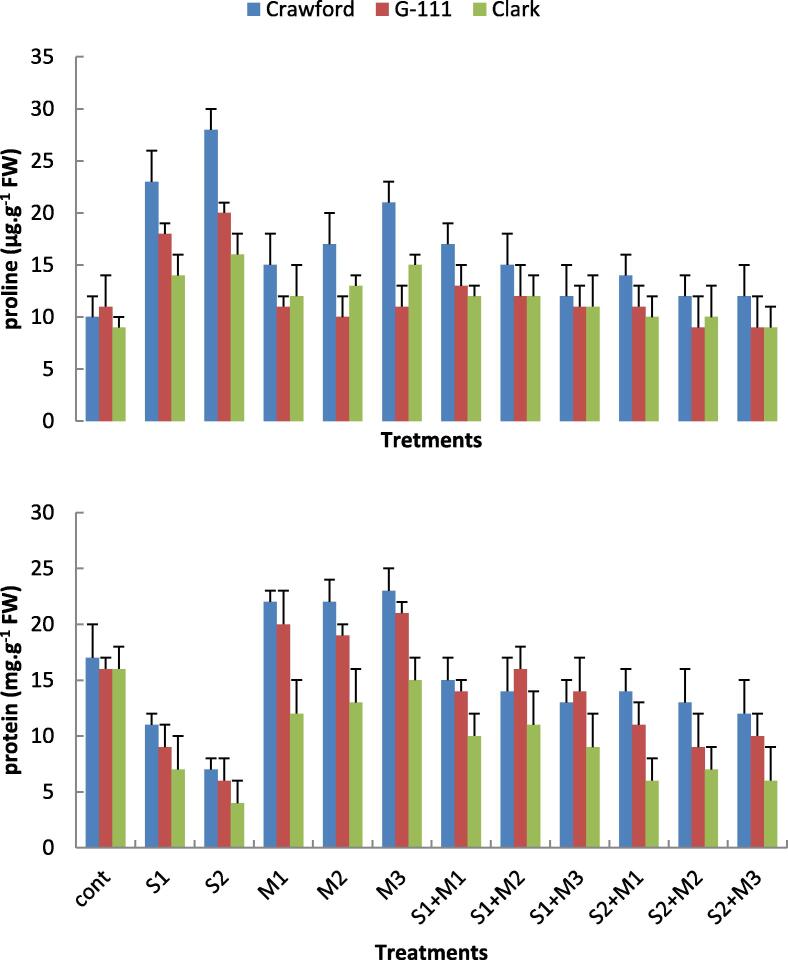
Effects of two salinity levels (S1 and S2) and three rhizobacterial isolates (M1, M2 and M3) on soluble protein (mg.g^−1^ FW) and free proline (μg.g^−1^ FW) of three cultivars of soybean leaves.

Soluble protein concentration (mg g^−1^ FW) in soybean plant leaves decreased by salinity treatments. The maximum decrease in soluble protein recorded under salinity stress was found in Clark cv., while the minimum decrease was recorded in Crawford followed by G-111 (Fig. 3). In rhizobacterial inoculated plants soluble protein content increased either in salt stressed or unstressed plants comparing with non-inoculated plants. In this regard, *B. subtilis* was the most effective isolate in increasing soluble protein in the absence of salinity stress. While in salt stressed plants *P. fluorescens* was more effective in improving soluble protein than the other isolates.

Proline concentration (*u*g g^−1^ FW) increased in leaves of salinity stressed plants as compared with control plants. In this connection, Crawford cv. recorded the highest values of proline content followed by G-111 then Clark. Moreover, the bacterial isolates caused an observed increase in proline content of salt untreated plants comparing with control, yet less than the effect of salinity. The collaboration of salinity stress and rhizobacterial isolates caused a predictable, though insignificant, change in proline content of soybean cultivars. At S1, proline concentration in G-111 and Clark cultivars was nearly unchanged with all isolates, while Crawford showed an observed increase in proline content. At S2 level proline concentration in any cultivar showed a significant response to the bacterial isolates (Fig. 3).

### 3.4 Antioxidant enzyme activities

**Fig. 4:**
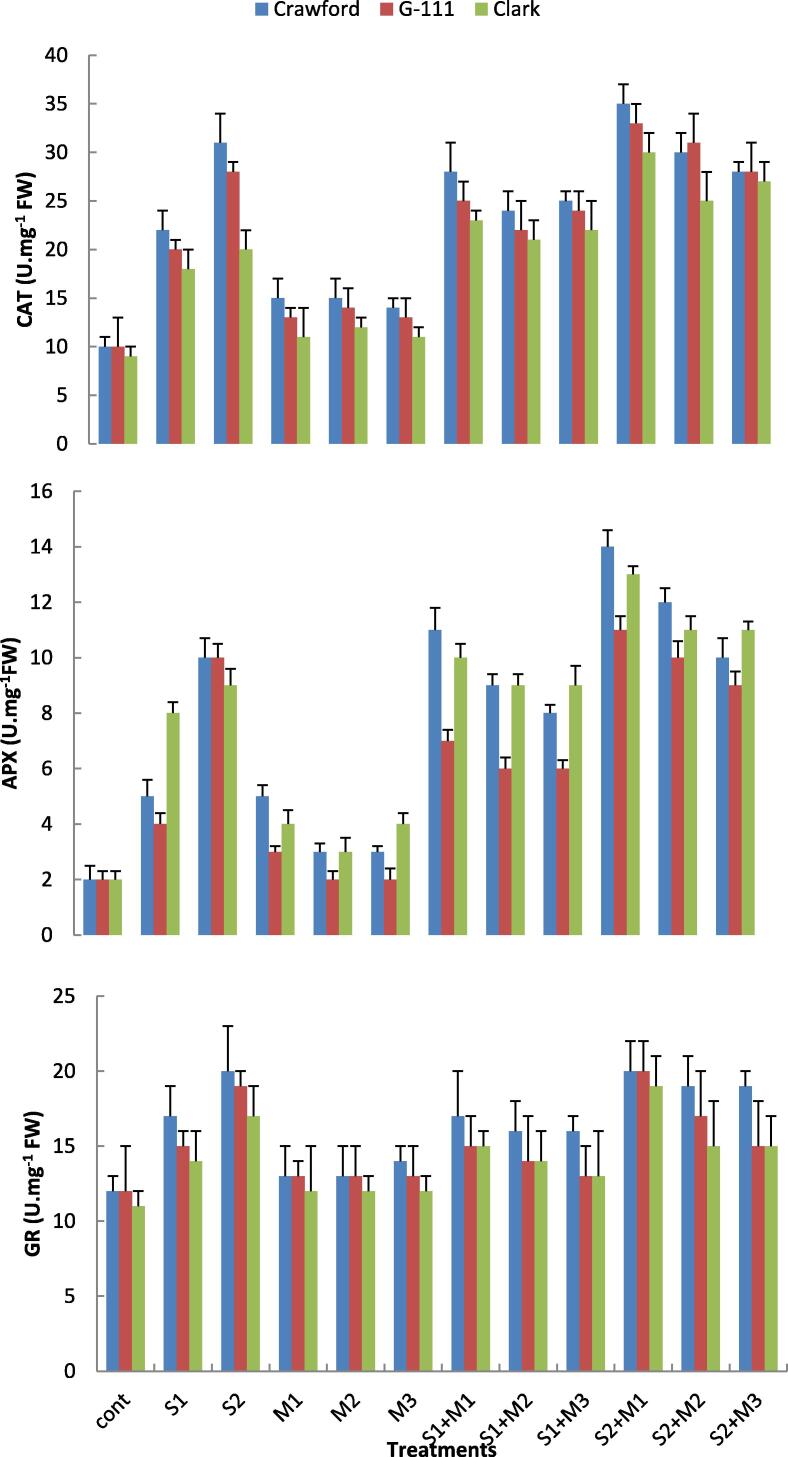
Effects of two salinity levels (S1 and S2) and three rhizobacterial isolates (M1, M2 and M3) on catalase (CAT) ascorbate peroxidase (APX) and glutathione reductase (GR) activities of three cultivars of soybean leaves.

Statistical analysis revealed that all soybean cultivars displayed a marked increase in the activity of enzymes CAT, APX and GR as NaCl concentrations increased. However, there were observed differences in the activities of the antioxidant enzymes between the three cultivars with increasing NaCl concentrations. The activity of the antioxidant enzymes was higher in salt tolerant Crawford cultivar under all salinity levels; while the APX activity was higher in Clark cv. under S1 treatment. All cultivars showed nearly close values of antioxidant enzyme activities under the control treatment. However, under high salinity (S2), the increasing rate of GR activity was estimated around 67% for Crawford, 58% for G-111 and 54% for Clark cultivars as compared with control. Similarly, CAT activity increased by three-fold in salt tolerant Crawford and two-fold for the less tolerant cultivar Clark. In addition, APX activity improved by around five-fold for Crawford and four-fold for Clark under the same growing condition (Fig. 4). It was clear that all antioxidant enzyme activities of all cultivars improved with the application of bacterial isolates particularly at salt stress conditions. It was clear that *P. fluorescens* isolate was more effective in increasing the antioxidant enzyme activities under salt stress conditions than other isolates.

### 3.5 Molecular study

#### 3.5.1 SDS- protein electrophoresis

**Fig. 5:**
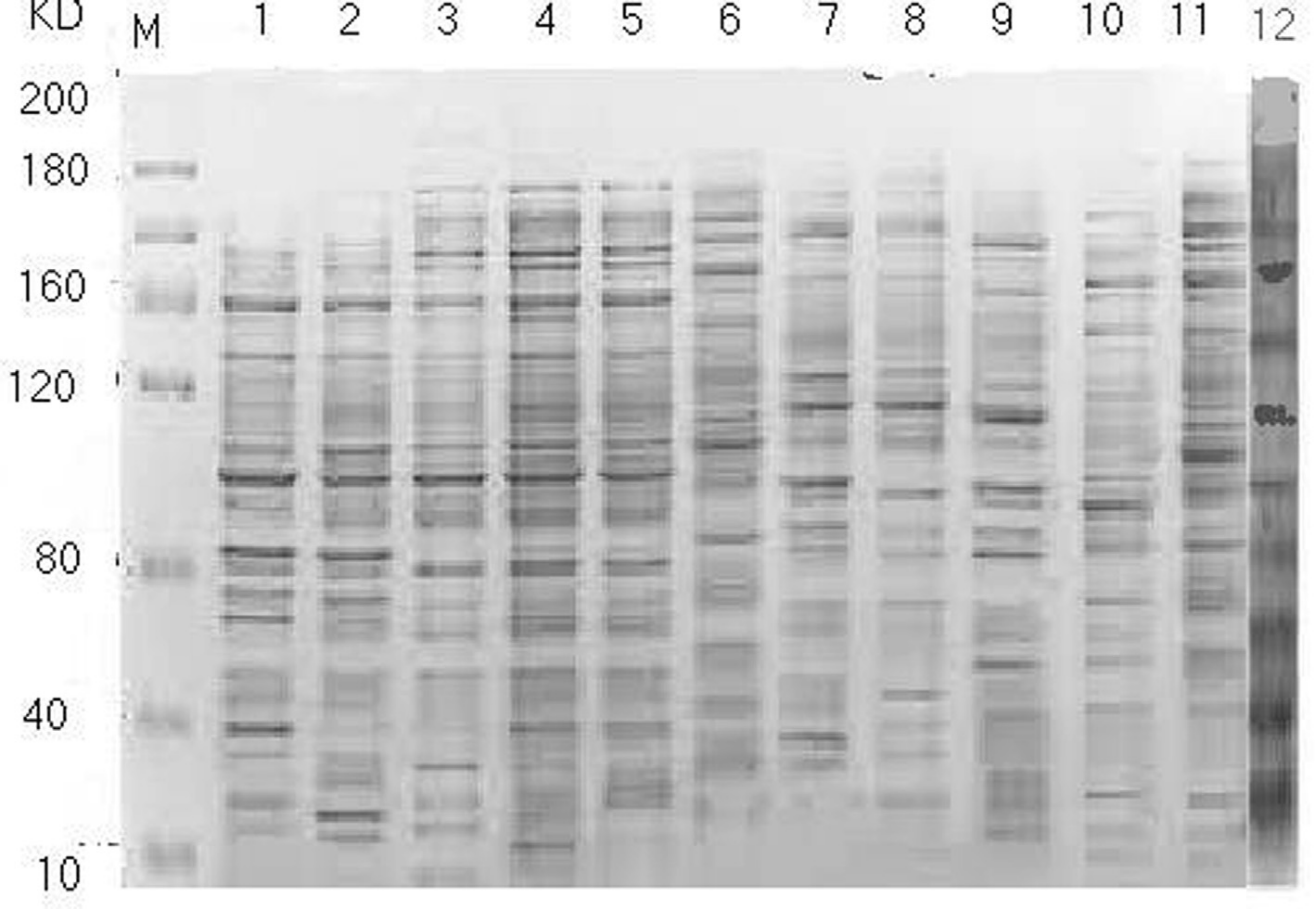
SDS-PAGE profile of total proteins from three cultivars of soybean leaves**.** No 1 = Control, 2 = S1, 3 = S2, 4 = Crawford + S1, 5 = Crawford + S2, 6 = G-111 + S1, 7 = G-111 + S2, 8 = Clark + S1, 9 = Clark + S2, 10 = S2 + M1, 11 = S2 + M2, 12 = S2 + M3, Treatments as in Table 1.

Protein banding patterns of the three soybean cultivars were revealed by SDS-PAGE and were shown in Table 2 and Fig. 5. According to the registered data, the total numbers of bands in all of the three cultivars were 20 bands with molecular weight (MW) ranged from 200 to 10KDa. There were some variations in the number of the obtained bands. The total number of bands among cultivars varied from 11 in Clark cultivar to 17 in Crawford cultivar. There are ten common bands that were found in all cultivars and were polymorphic while the rest were monomorphic bands. Bands with MW of 200, 170, 25 and 15KDa are present in cultivar Crawford and absent in all other cultivars, while band with MW about 45KDa considered as a marker for cultivar Clark. In conclusion, the present study recorded two common markers for soybean cultivars Crawford, Giza-111 and Clark.

**Table 2:** SDS-PAGE of total proteins extracted from the three soybean cultivars.

Soybean cultivars			
MW (kDa)	Crawford	G-111	Clark
200	+	–	–
170	+	–	–
160	+	+	+
150	+	+	+
140	+	+	–
130	+	+	+
120	+	+	+
100	+	+	–
90	+	+	+
80	+	+	+
70	+	+	+
60	–	–	–
50	+	+	+
45	–	–	+
40	+	+	–
30	+	+	+
25	+	–	–
20	–	–	–
15	+	–	–
10	+	+	+
Total + fragments	17	13	11
Fragment%	85	65	55

#### 3.5.2 ISSR-PCR primers

The genetic and phylogenetic diversity relationships among the three tested soybean cultivars were demonstrated using the following three arbitrary primers: B04 (GGACTGGAGT), B06 (TGCTCTGCCC) and B12 (CCTTGACGCA). Data in Table 3 revealed that all primers showed protein banding patterns on the gel with different polymorphism percentages. A total of 54 alleles were separated by electrophoresis and were observed on the agarose gel, ranging from 200 to 450 bp in size. Out of the 54 fragments, 38 + fragments were present, out of them about 9 were polymorphic (presenting 32.7%) and the rest (67.3%) were monomorphic, across the 3 used cultivars (Table 3). The maximum number of bands (24) and 4 polymorphisms were produced with primer B12 compared to the other ISSR primers (B04 and B06), while the less number of bands (15) and polymorphism (18%) were observed with B04 primer. The highest number of bands was generated in c.v. Crawford (17 bands), while c.v. Clark produced the lowest number (11 bands) as shown in Table 3. It was clear that values of polymorphism level among the studied cultivars were low. The higher ratio of polymorphism was shown to be random; however, 20 bands were distinguished as markers related to salt stress conditions (17 positive and 3 negative) (Table 2).

**Table 3:** DNA polymorphism in three soybean cultivars using PCR with three random primers.

Bp		Crawford	G-111	Clark	Total bands	PolymorNo.	Polymor (%)
450	B04	+	–	+			
350		+	+	+			
245		–	+	+	11	2	18.2%
220		+	+	–			
200		+	+	–			
470	B06	+	+	+			
365		+	+	+	11	3	27.2%
320		+	–	+			
285		+	+	+			
200		–	–	–			
750	B12	+	–	–			
680		+	+	+			
645		+	+	–			
610		+	+	+	18	4	22.2%
515		–	+	–			
470		+	–	+			
315		+	+	+			
200		+	+	+			
Total +		15	13	12	40	9	22.5%

**Fig. 6:**
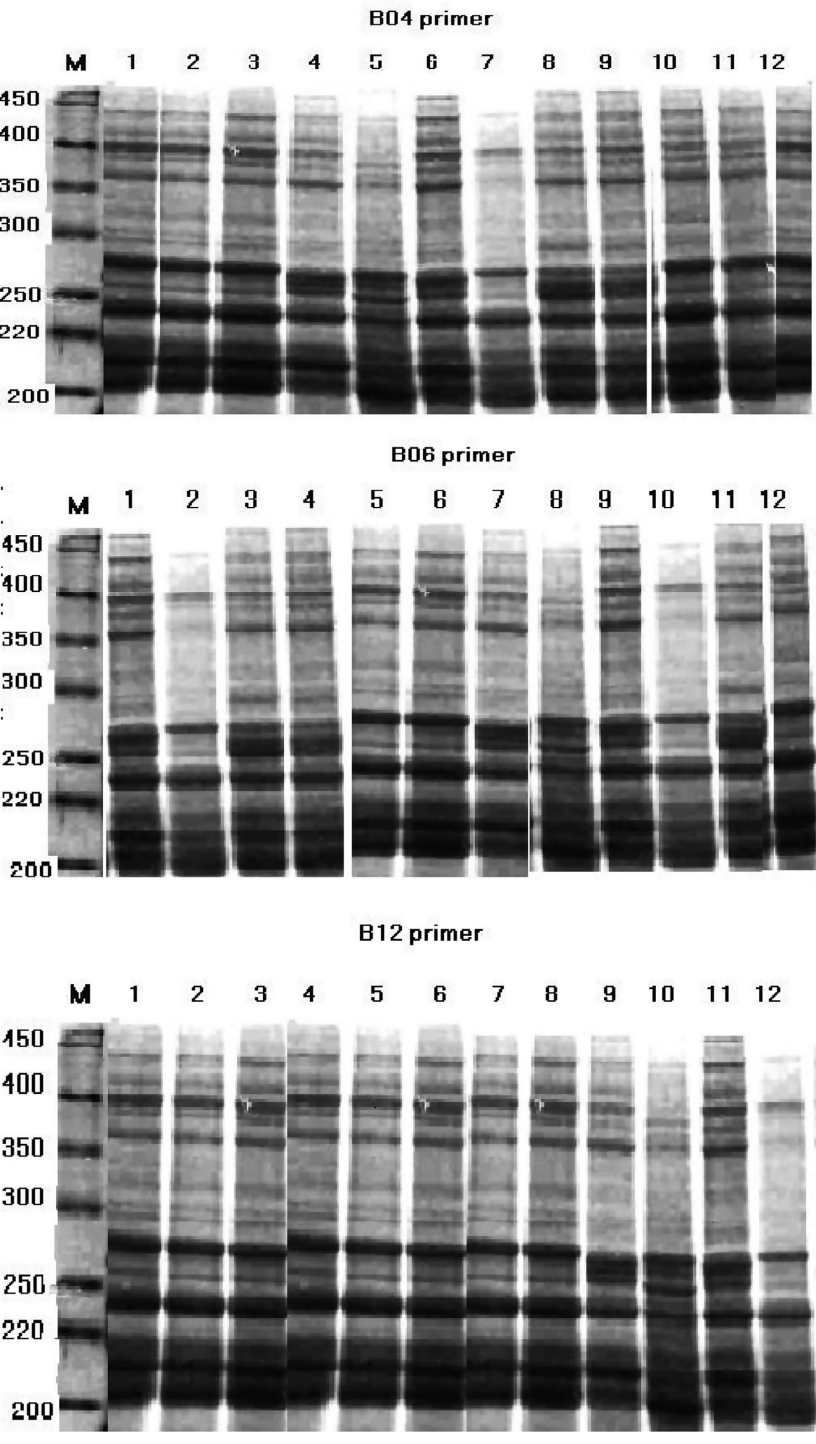
DNA banding patterns of soybean leaves generated by different ISSR-PCR primers. M; 1Kd DNA Ladder. No 1 = Control, 2 = S1, 3 = S2, 4 = Crawford + S1, 5 = Crawford + S2, 6 = G-111 + S1, 7 = G-111 + S2, 8 = Clark + S1, 9 = Clark + S2, 10 = S2 + M1, 11 = S2 + M2, 12 = S2 + M3, Treatments as in Table 1.

Once B04 primer was used, it resulted in an amplified fragment in all cultivars. This primer also produced an amplified fragments in salt tolerant (Crawford, 4 bands) and moderate (G-111, 4 bands) cultivars and salt sensitive Clark (3 bands). The outcome from ISSR analysis using B04. B06 and B12 are demonstrated in Fig. 6. Two amplified DNA fragment of 200 and 450 bp were produced in all cultivars with these primers. From the ISSR profiles produced by the tested primers some bands were absent in the sensitive. Clark cv. Using primer B12, an amplified fragments were created only in salt tolerant Crawford cv., while absent in other cultivars.

## 4 Discussion

Salinity stress is an important environmental factor that can prevent many crop species from achieving their full inherited genetic strength, therefore, salinity stress often causes several limitations in growth and physiology of soybeans (Sadak et al., 2020). The present study showed that soybean plants were able to grow normally at low levels of salinity (such as 200 mM NaCl and lower) since growth parameters were not affected significantly at such concentrations; but when plants were exposed to relatively higher salt concentrations, all cultivars experienced a significant reduction in their heights and biomass. However, the response of the three tested cultivars to salinity was found to be different. Salt tolerant cultivar Crawford showed better growth than moderate tolerant G-111 cultivar or salt sensitive Clark. The decline in plant growth and biomass under abiotic stress conditions including salinity has been reported in several grain legumes (Taïbi et al., 2016, Hernandez, 2019, Sadak et al., 2020;). Growth reduction of salt-stressed plants was attributed to the inhibition of cell division and elongation (Mo et al., 2020).

Recorded data showed that salinity stress resulted in a significant decrease in growth characters as compared with unstressed control plants. Chlorophyll content was also decreased in salt treated plants. Similarly, the decline in growth parameters were found in many plant species grown under saline conditions (Kaya et al., 2013). The negative effect of salinity on growth parameters could be ascribed to the toxic effect of salinity or to the high osmotic pressure developed by salt, at which soybean plants be unable to uptake water and nutrients. In addition, salinity stress often generate an excessive reactive oxygen species (ROS), which cause cell toxicity, membrane destruction and cell death (Mo et al., 2020).

Our results showed that the growth of salt-stressed soybean plants was improved in the presence of rhizobacterial isolates. Similar results were obtained by Metwali et al. (2015) who showed an increase in plant growth parameters of bean by the inoculation with *P. fluorescens* and *P. putida* under salinity conditions. Several explanations have been suggested to understand the mechanism by which rhizobacteria can improve plant tolerance to salt stress; one mechanism is the production of growth hormones, resulted in increased root growth, leading to higher uptake of nutrients and improving the whole plant growth under salt stress conditions (Kumar et al., 2016). It is well known that, under saline conditions, root-colonizing bacteria often produce growth regulator chemicals including auxin that helps maintaining the growth of plant roots under salt stress conditions, and can contribute to sustaining leaf growth rate (Albacete et al. 2008). Besides the ability of these microbes to limit or stop transport of toxic ions such as Na^+^ and Cl^-^ from plant roots to shoots (Golpayegani and Tilebeni, 2011). They reported that *Pseudomonas* Sp. and *Bacillus lentus* relieved the harmful effect of salt stress on growth parameters, photosynthesis process, mineral uptake and content as well as antioxidant enzymes activity in basil plants. In this regard, Kumar et al. (2016) reported that the rhizobacteria promoted root branching and increased secretion of flavonoids and oligosaccharides in chickpea plants.

In agreement with earlier results of Taibi et al. (2016) on *Phaseolu*s *vulgaris* and by Rahmawati et al. (2014) on Glycin max plants, results recorded in this study showed that chlorophyll *a* and chlorophyll *b* substantially decreased under salinity stress conditions. The decline in chlorophyll content under salinity stress conditions has been referred as a distinctive sign of oxidative stress induced at salinity conditions (Taibi et al., 2016) and was ascribed to the obstruction of chlorophyll formation and to the stimulation of chlorophyll degradation by the enzyme chlorophyllase. Thus, the decrease in chlorophyll contents could be attributed to either slow synthesis or fast degradation of chlorophyll pigments.

Previous studies showed that, several chemicals including soluble proteins and proline involved in osmotic regulation of salt stressed plants, thus playing an important role in the tolerance of plants under salinity stress conditions (Meena et al., 2019), and may be used as a positive approach to reduce the toxicity of Na^+^ ions (Tabssum et al., 2018). In the present study, salt treatments, particularly with S2, resulted in a remarkable decline in protein content, but proline concentration increased significantly in salt-stressed plants compared to the normal control. These results were similar to that of Sadak et al. (2010). In this connection, Ramezani et al. (2011) reported that, the decline of soluble protein content under abiotic stress was accompanied by accumulating nitrogenous amino acids and free proline in the cytoplasm thus playing an important role in the osmotic balance of plants. Therefore, accumulation of proline and other amino N are considered clear indicators for tolerance to salt stress (Ramezani et al., 2011). These assumptions were confirmed with the results of the present study. The enhanced proline content in Crawford soybean cultivar under salt stress indicates that proline accumulation considers as an outstanding tool to decrease the osmotic potential in this cultivar. These findings support the idea that proline accumulation is a part of physiological responses of plant to salinity stress (Meena et al., 2019).

Plants often produce some antioxidant enzymes to avoid oxidative damage under stress conditions and keep concentrations of the reactive oxygen species (ROS) within a limited and narrow functional range (Akyol et al., 2020). It is well known that antioxidant enzymes such as catalase (CAT), ascorbic acid peroxidase (APX) and glutathione reductase (GR) activities considerably decrease the levels of superoxide and hydrogen peroxide in salt-stressed plants (El-Beltagi et al., 2020). Activities of the antioxidant enzymes CAT, APX and GR were found to be higher in the salt tolerant cultivar Crawford than in the salt sensitive cultivar Clark under NaCl treatments. As reported by Rao et al. (2013), the higher activity of antioxidant enzymes caused a decrease in lipid peroxydation under salinity conditions. In addition, a previous study by El-Beltagi et al. (2020) showed that CAT and APX enzymes were highly active antioxidants in avoiding cell collapse or damage. Thus, both enzymes showed a significant importance in regulating H_2_O_2_ at intracellular levels (Sofo et al., 2015)). They reported also that APX enzyme enhanced protection against oxidative stress conditions. Data of this study illustrated that APX activity increased with increasing salinity stress in all cultivars, however, its activity was significantly higher in the tolerant cultivar Crawford under S2 level, particularly with rhizobacterial isolates. It was remarkable that salinity-induced APX activation in the tolerant cultivar Crawford was associated with a higher increase in CAT activity. Therefore, APX and CAT might have an equal effect in detoxifying H_2_O_2_ under high salinity stress in the tolerant cultivar. These results confirm early results of Taïbi et al. (2016) who reported that APX and CAT together play a vital protective role in plant tolerance under salinity stress conditions. Increasing GR activity was found also to be involved in salt tolerance of different crops (Kusvuran et al., 2016). The capability to synthesize amino acids rich in sulphur was decreased as a limiting factor for oxidative stress derived from abiotic stress conditions (Mukwevho et al., 2014). Our results suggest that the less tolerant cultivars such as G-111 and Clark showed a less active ascorbate–glutathione cycle under high salinity stress conditions. In this regard, Taha et al. (2020) showed that enhancing salt tolerance of soybean by potassium treatment was established through enhancing the antioxidant defense system under saline conditions.

Data illustrated that the three selected primers (B04, B06 and B12) displayed banding profiles with low polymorphism (Fig. 6) and each primer produced fingerprint profile exclusive to each cultivar; therefore, primers can be used individually to classify the tested soybean cultivars. Two from the three studied primers, B06 and B12 produced clear multiplex banding profiles. These findings were in agreement with those reported by Maryam et al. (2015) on *Vicia faba*. Their study illustrated that primers which based on CT/CT and CC/TT dinucleotide core can produce good banding profiles. These results were explicated by Riaz et al. (2018), who concluded that dinucleotide primers were more appropriate for amplifying ISSRs and (CT) dinucleotide repeats are most abundant in many plant species. The low percentage of polymorphism found in this study was also reported in earlier investigations (Wang et al., 2011, Dagnew et al., 2014). The low value of polymorphism might be attributed to inherently constricted genetic base and also to out crossing made in pollination of soybean crop.

Eighteen bands out of 40 were valued as positive or negative markers linked to the salt stress tolerant cultivar (Crawford). These 18 bands were produced by all primers which produced both positive and negative markers as shown in Fig. 6. Fifteen positive and three negative bands were generated in the salt tolerant Crawford, while the less tolerant Clark produced 12 positive and 6 negative bands. The salt tolerant cultivar considered as a promising line with high confidence established on their genetic evidences rather than phenotypic structure. This conclusion agreed with those of (Rasha, 2013, Metwali et al., 2015) who reported that the efficiency of ISSR-PCR to improve the documentation of tolerant crops to abiotic stress conditions.

In the present study it seemed that genetic resemblance at ISSR levels was able to identify the genetic relationship between soybean cultivars. The highest similarity values were registered among G-111 and Clark cultivars in together, which proved that these two genotypes are closely related to each other (Fig. 5, Fig. 6). This finding was revealed via their reaction to salinity stress condition, however, the less value of similarity was registered among both cultivars and Crawford. These findings indicated that the tested cultivars were genetically varied and distant genotypes in their response to salt stress. Based on results of the present study, it is essential to evaluate the importance of molecular markers in screening and selecting the high salt-stress tolerant genotypes. Our study verified the effectiveness of genetic relationships between closely related genotypes. These observations approved strongly those of Das and Sahoo (2020).

## 5 Conclusion

In conclusion, salinity stress at 200–400 mM decreased final germination percent, growth parameters, chlorophyll and soluble protein contents in leaves. The decrease in shoot fresh weight was about 27.4% at 200 mM of NaCl and about 45.5% at 400 mM. Chlorophyll was reduced by about 68.7% in Crawford and 85.3% in Clark. In addition, Protein fragments were 17, 13 and 11 in Crawford, G-111 and Clark, respectively, indicating that Crawford was the most tolerant cultivar to salinity conditions while Clark was the least. The inoculation with some bacterial strains (*Pseudomonas fluorescens*, *P*. *putida* and *Bacillus subtilis*) alleviated the harmful effects of salinity stress on soybean cultivars. Proline and antioxidant enzymes increased with increasing salt concentration, particularly in salt tolerant cultivar. The results affirm that molecular studies using PCR-RAPD markers and ISSR primers are important to recognize the alleles associated with the salt stress in soybean cultivars.

## Declaration of Competing Interest

The authors declare that there is no conflict of interests regarding the publications of this paper.
